# Profiles of resources and body image in health and illness: A comparative study among females with rheumatoid arthritis, females with breast cancer and healthy controls

**DOI:** 10.1002/brb3.1488

**Published:** 2019-12-04

**Authors:** Marcin Rzeszutek, Małgorzata Pięta, Marek Huzar

**Affiliations:** ^1^ Faculty of Psychology University of Warsaw Warsaw Poland

**Keywords:** body image, breast cancer, resources, rheumatoid arthritis

## Abstract

**Background:**

The aim of this study was to examine whether or not profiles of resources (i.e., a multifaceted picture that simultaneously includes different types of resources), as described by the conservation of resources (COR) theory, and profiles of body image (i.e., a multidimensional picture that simultaneously includes different aspects of body image) differ between females that represent two clinical samples (rheumatoid arthritis [RA]; breast cancer [BC]) and a healthy control group.

**Method:**

The sample comprised 328 females, including 141 women with RA, 102 with a BC diagnosis, and 85 healthy women as a control group, and was collected from the general population. To measure the level of COR resources in each participant, we used the COR evaluation questionnaire (COR‐E). Participants' body image was assessed with the aid of the Multidimensional Body‐Self Relations Questionnaire (MBSRQ).

**Results:**

A discriminant analysis revealed that females from the clinical groups differed with respect to their profiles of some resources and body image when compared to those of the healthy control group. In addition, we found differences in body image evaluations between women with RA and women with BC.

**Conclusions:**

Women with RA or BC differ substantially with respect to their subjectively assessed resources and body image when compared to women with no chronic diseases. Therefore, psychological counselling designed for females with RA or BC should be employed to help them restore the aspects altered by their respective illnesses.

## Graphical abstract

The aim of our study was to examine whether profiles of resources, as described by the conservation of resources theory (COR), and body image differ between females representing two clinical samples (rheumatoid arthritis, RA; breast cancer, BC) compared to healthy control group. Secondly, we aimed to investigate the potential differences between profiles of resources and body image within aforementioned clinical samples only. Until now, the vast majority of studies on coping with various chronic disorders have focused predominantly on the transactional model of stress and coping (*Stress, appraisal and coping*. New York: Springer, 1984). Taking this fact into an account, but also several limitations of aforementioned model in depicting the process of coping with disease, in our study we referred to a much understudied theory with this regard, that is conservation of resources theory (COR; *American Psychologist*, 44, 1989, 513). However, the majority of studies on COR theory have been conducted in nonclinical settings, so little is known about the application of this model in clinical environment among patients illness‐related distress. Thus, in our study we wanted to compare the COR resources evaluation among two clinical samples of female participants, that is rheumatoid arthritis (RA) and breast cancer (BC).

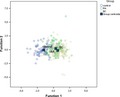

## 1 INTRODUCTION

Chronic illness is a phenomenon that is growing in prevalence, affecting an increasing portion of the population all over the world (World Health Organization [WHO], 2018). Chronic illness can be very stressful and is associated with major functional limitations (e.g., arthritis; Shih, Hootman, Strine, Chapman, & Brady, 2006) and even traumatic experiences that pose a real threat to life (e.g., cancer; Kangas, Henry, & Bryant, 2005). In other words, chronic disease can induce a profound change in patients' lives and well‐being, and thus, appropriate stress‐coping mechanisms are necessary to help patients adapt to the sometimes overwhelming situation of coping with an ongoing illness (e.g., Dempster, Howell, & Mccorry, 2015). Until now, the vast majority of studies on coping with chronic disorders have focused on the transactional model of stress and coping (Lazarus & Folkman, 1984; for reviews, see also Kato, 2013; Moskowitz, Hult, Bussolari, & Acree, 2009). Taking this into account, it is important to note that there are several limitations to the aforementioned model in depicting the process of coping with disease (e.g., Skinner, Edge, Altman, & Sherwood, 2003); in our study, we focus on the massively understudied conservation of resources (COR) theory (Hobfoll, 1989).

Conservation of resources theory focuses on the sociocultural context of stress and coping, wherein the central attention is shifted from a subjective appraisal of stress and coping (Lazarus & Folkman, 1984) to the objective resources defined as things that an individual currently possesses and values (e.g., objects, states, or conditions) or aims to achieve, maintain, and protect in the future (Hobfoll, 1989). Therefore, in COR theory, stress and coping processes can be operationalized more specifically and are associated with an actual loss of resources or the threat of losing them. The majority of studies on COR theory have been conducted in nonclinical settings (e.g., Hobfoll, Johnson, Ennis, & Jackson, 2003; Jin, McDonald, & Park, 2016; van Woerkom, Bakker, & Nishii, 2016), and thus, little is known about the application of this model in a clinical environment (e.g., Banou, Hobfoll, & Trochelman, 2009; Dirik & Karanci, 2010). Thus, in our study, we evaluate and compare the COR resources among two clinical samples of female participants, those with rheumatoid arthritis (RA) and those with breast cancer (BC). We chose these clinical entities mainly due to the biological links between them (i.e., there is a heightened risk of BC in RA females; see Tian, Liang, Wang, & Zhou, 2014). Moreover, it remains unknown as to how clinical and nonclinical samples differ in their resources' perception. Thus, we also wanted to compare resource evaluations among the clinical samples to those of a healthy female control group.

Rheumatoid arthritis is one of the leading debilitating chronic diseases worldwide and is characterized by joint destruction, chronic pain, incremental disability, and heightened mortality compared to that of the general population (Smolen, Aletaha, & McInnes, 2016). The aforementioned factors are linked with persistent psychological distress (Rzeszutek, Oniszczenko, Schier, Biernat‐Kałuża, & Gasik, 2016), poor quality of life (Matcham et al., 2014), and, in the long run, various RA‐related psychiatric disorders (Nicassio et al., 2012). Nevertheless, many researchers have noticed various benefits related to coping with RA. The literature on coping with RA, although vast, is very heterogeneous with regard to conclusions and is based mainly on Lazarus and Folkman's (1984) model (e.g., Conner et al., 2006; Newth & Delongis, 2004). Until now, only Dirik & Karanci (2010) had observed that resource loss, as described by COR theory, was the strongest predictor of depression of all studied psychosocial variables, and this effect was most visible among female RA patients.

Along with RA, BC is one of the most prevalent health problems worldwide, being the second most frequently detected type of cancer in the world and the first most frequently detected among women (WHO, 2018). Women with BC face multidimensional psychological distress stemming from its threat to life (Saboonchi et al., 2014) along with a significant drop in their quality of life and a decline in their psychosocial functioning (Syrowatka et al., 2017). Like in RA patients, appropriate coping abilities can be crucial in adapting to this disease, which are linked with less anxiety, lower levels of depression, and an increased quality of life (Arnaboldi, Riva, Crico, & Pravettoni, 2017). However, until now, only Banou et al. (2009) had demonstrated the role of interpersonal resources from COR theory in diminishing cancer‐related distress in women with BC.

The second reason we compared these two clinical entities is their relationship with one psychological variable that plays a major role in coping with them—that is, body image. *Body image* is a multidimensional term that encompasses the thoughts, beliefs, emotions, and behaviors associated with an individual's physical appearance (Cash & Pruzinsky, 2002). In RA patients, body image distortions, which are very prevalent, are related to elevated pain and functional limitations, all of which have been observed mainly among female RA patients (Bode, Taal, Heij, & Laar, 2010; Gutweniger, Kopp, Mur, & Gunther, 1999). Likewise, body image distortions in BC are the strongest predictor of cancer‐related distress and the deterioration of social functioning in females with BC (Carver et al., 1998; Thomas & Usher, 2009). It has been determined that in females with RA (Alleva et al., 2018; Bode et al., 2010) and those with BC (Fobair et al., 2005; Harcourt & Rumsey, 2019), body image is shaped to a greater extent by psychosocial factors than by illness‐related factors, but no studies on the differences between COR resources and body image in these patient groups have been conducted thus far.

### 1.1 Current study

Taking the abovementioned research gaps into consideration, the aim of our study is twofold. First, we want to examine whether or not the profiles of resources and body image differ between the females that represent different clinical samples (i.e., RA and BC patients) and a healthy control group. Second, we aim to investigate the potential differences between the profiles of resources and body image within the aforementioned clinical samples. We formulated four hypotheses:

Hypothesis 1 Clinical samples of the females differ with respect to the profiles of resources, as described by the COR theory, from the healthy control group of females.

Hypothesis 2 Females with RA differ with respect to the profiles of resources, as described by the COR theory, from female participants with BC.

Hypothesis 3 Clinical samples of the females differ with respect to body image profiles from the healthy control group of females.

Hypothesis 4 Female participants with RA differ with respect to body image profiles from female participants with BC.

## 2 METHOD

### 2.1 Participants and procedure

The study sample comprised 328 females, including 141 women with RA, 102 with a BC diagnosis, and 85 healthy women (i.e., with no chronic diseases), and was collected from the general population. The female participants with RA were recruited from the patients of the National Institute of Geriatrics, Rheumatology, and Rehabilitation in Warsaw, Poland. The female participants with BC were recruited from the Magodent Oncology Hospital in Warsaw. The healthy control group was recruited from a nonclinical population among the students of various Warsaw universities.

The study subjects filled out paper‐and‐pencil questionnaires and participated in the study voluntarily, and thus, no remuneration was provided for participation. In cases of clinical samples, the eligibility criteria encompassed being 18 years of age or older and having a confirmed medical diagnosis of RA or BC, which was screened by medical doctors working in the hospitals where the research was conducted. The exclusion criteria for clinical samples included cognitive impairment, vision loss, or major joint changes that limited writing skills among RA patients and a poor emotional state among BC patients, which was diagnosed by clinical psychologists. For the nonclinical sample, the inclusion criteria encompassed being 18 years of age or older and self‐declaring no chronic illnesses. The research project was approved by the ethics committee. Table 1 presents the sociodemographic characteristics of all study participants with statistical tests for differences between the groups.

**Table 1:** Sociodemographic variables in the studied samples

Variable	Group			*F*/*χ* ^2^	*p*
	Control (*N* = 85)	RA (*N* = 141)	BC (*N* = 102)		
Age in years (*M* ± *SD*)	25.89 ± 10.19	57.26 ± 12.36	54.45 ± 15.02	152.94	.001
Marital status					
Married	53 (62.4%)	66 (64.7%)	79 (56.0%)	2.57	.277
Single	32 (37.6%)	34 (33.3%)	62 (44.0%)		
Education					
Elementary	0 (0%)	5 (4.9%)	23 (16.3%)	26.41	.001
Secondary	57 (67.1%)	49 (48.0%)	73 (51.8%)		
Higher education	28 (32.9%)	48 (47.1%)	45 (31.9%)		
Employment					
Full employment	28 (32.9%)	20 (19.6%)	51 (36.2%)	123.45	.001
Unemployed	54 (63.5%)	0 (0%)	10 (7.1%)		
Illness allowance	1 (1.2%)	6 (5.9%)	25 (17.7%)		
Retired	2 (2.4%)	12 (11.8%)	55 (39.0%)		
Place of residence					
Village, small town up to 20 thousand residents	13 (15.3%)	13 (12.7%)	44 (31.2%)	51.03	.001
City 21 to 100 thousand residents	2 (2.4%)	2 (2.0%)	27 (19.1%)		
City 101 to 500 thousand residents	1 (1.2%)	2 (2.0%)	19 (13.5%)		
City over 500 thousand residents	67 (78.8%)	21 (20.6%)	49 (34.8%)		
Lack of permanent residence	1 (1.2%)	0 (0%)	1 (0.7%)		

Abbreviations: *F*, analysis of variance; *M*, mean value; *SD*, standard deviation; *χ* ^2^, Pearson chi‐squared test of independence.

The groups differed in terms of participants' age, education, employment, and place of residence. Post hoc analysis revealed that participants from the control group were significantly younger than RA patients, *p* < .001, and BC patients, *p* < .001. The number of married participants was significantly higher in the RA group than in control group, *χ* ^2^(1) = 4.87, *p* < .05, and the BC group, *χ* ^2^(1) = 11.28, *p* < .01. Education level was lower in the BC group than in the control group, *χ* ^2^(1) = 16.04, *p* < .001, and the RA group, *χ* ^2^(1) = 10.40, *p* < .01. Education level in the RA group was also significantly better than in the control group, *χ* ^2^(1) = 11.31, *p* < .01. The number of participants without regular unemployment was significantly bigger in the control group than in the RA group, *χ* ^2^(3) = 54.72, *p* < .001, and the BC group, *χ* ^2^(1) = 100.69, *p* < .001. The number of participants living on the village or in a small town was significantly bigger in the BC group, and the number of participants living in cities with over 500 thousand residents was significantly bigger in the control group, *χ* ^2^(3) = 46.30, *p* < .001.

### 2.2 Measures

To assess the level of subjectively possessed resources among the participants, we used the COR evaluation questionnaire (COR‐E; Hobfoll, Lilly, & Jackson, 1992) in the Polish adaptation of Dudek et al. (2006). The COR‐E consists of two parts (A and B), each with 40 items. In part A, participants rated the extent to which they attach importance to several resources, represented by objects, states, or conditions, while in part B, they rated the extent to which they possess these resources. The items in parts A and B refer to the following resources: hedonistic and vital resources, spiritual resources, family resources, economic and political resources, and power and prestige resources. Higher scores on the COR‐E indicate higher levels of resources.

Participants' body image was evaluated using the Multidimensional Body‐Self Relations Questionnaire (MBSRQ), constructed by Thomas Cash (2000) and adapted to Polish by Brytek‐Matera and Rogoza (2015). We paid an appropriate nominal fee for the use of the MBSRQ in this particular research. The MBSRQ consists of 10 scales that evaluate several elements associated with body image: the appearance evaluation scale, the appearance orientation scale, the fitness evaluation scale, the fitness orientation scale, the health evaluation scale, the health orientation scale, the illness orientation scale, the body areas satisfaction scale, the overweight preoccupation scale, and the self‐classified weight scale. The higher the results in each subscale, the more positive the assessment of particular aspects of body image was reported by a participant.

### 2.3 Data analysis

The introductory portion of this study's statistical analysis comprised descriptive statistics between the analyzed variables. The principal part of the analysis was performed with the use of a discriminant analysis (Mclachlan, 2004). Discriminant analysis is a statistical analysis used to verify associations between interval explanatory variables and categorical outcome variables. It creates variates called discriminant functions that consist of interval explanatory variables. These variates are then used to discriminate between the outcome variable categories, which allows for an analysis of between‐group differences regarding the levels of analyzed variables and the differences between them. Group membership was analyzed using the grouping variable; the levels of resources and body image dimensions were analyzed as independent variables using two separate statistical models. The control group and both clinical groups significantly differed in terms of age and other differences, including education and employment, which were consequences of this age variability. The differences between groups in terms of demographic variables were verified with the use of a one‐way ANOVA and Pearson's *χ* test for independence. The mean age of participants from the control group was lower than the mean values of age in both clinical groups. More participants from the control group had received secondary education, were unemployed, and lived in cities with over 500 thousand residents.

The difference in participants' ages was controlled for in both statistical models. The choice of discriminant functions was based on Wilks's lambda test (Mclachlan, 2004), which allows for the deciding of how many extracted variates discriminate between the categories of outcome variables. The interpretation of the acquired discriminant functions, when significant, was based on the values of standardized canonical discriminant function coefficients. A positive correlation meant there was a positive association between the explanatory interval variable and the extracted variate. A positive and a negative correlation between interval variables in the same variate meant that the difference between the two interval variables discriminated between the categories of the outcome variable. The values of the functions at the group centroids were depicted on graphs to make the interpretation easier. A partial eta‐squared (*η* ^2^) effect size measure was used to make the interpretation of effect sizes possible. Following the classic guidelines of Cohen (1988), the values of *η* ^2^ should be interpreted as small if the values are less than .06, medium if the values are in the range from .06 to .14, and large if the values are greater than .14.

## 3 RESULTS

Table 2 presents the descriptive statistics for all analyzed interval variables in the study samples.

**Table 2:** Descriptive statistics and pearson correlation coefficients between analyzed variables in the whole study sample (*N* = 328)

Variables	*M*	*SD*	*S*	*K*
1. Hedonistic and vital resources	146.35	57.93	−0.30	−0.26
2. Spiritual resources	104.75	33.76	0.58	3.37
3. family resources	153.72	50.40	−0.40	−0.82
4. Economic and political resources	111.03	45.08	0.82	0.86
5. Power and prestige resources	40.00	24.16	0.32	0.92
6. Appearance evaluation	3.30	0.77	0.80	0.87
7. Appearance orientation	3.35	0.61	0.32	0.46
8. Fitness evaluation	3.02	0.83	−0.01	−0.13
9. Fitness orientation	3.06	0.74	0.77	0.69
10. Health evaluation	3.22	0.66	−0.14	0.36
11. Health orientation	3.40	0.58	−0.30	0.46
12. Illness orientation	3.39	0.73	0.13	0.36
13. Overweight preoccupation	2.58	0.83	0.13	−0.59
14. Body areas satisfaction	3.28	0.77	0.41	0.97
15. Self‐classified weight scale	3.27	0.76	0.20	0.80

Abbreviations: *K*, kurtosis; *M*, mean value; *S*, skewness; *SD*, standard deviation.

The skewness and kurtosis values fell between −1 and 1, and thus, the use of parametric statistical methods was appropriate. To verify Hypothesis 1 and Hypothesis 2, a discriminant analysis was performed. The levels of all five types of resources and participants' ages were analyzed as independent variables. Table 3 presents the values of the standardized canonical discriminant function coefficients. Wilks's lambda test revealed that only the first function, *χ* ^2^(12) = 200.07, was statistically significant (*p* < .001). The second function, *χ* ^2^(5) = 7.43, was statistically insignificant (*p* > .05), which means only the first discriminant function discriminated between the analyzed groups.

**Table 3:** Standardized canonical discriminant function coefficients for the level of resources

Level of resources	Function 1	Function 2
Hedonistic and vital resources	−1.32	0.24
Spiritual resources	0.01	−0.57
Family resources	0.82	0.11
Economic and political resources	0.27	1.06
Power and prestige resources	0.27	−0.14
Age	0.70	0.56

Note

Positive values of standardized canonical discriminant function mean that the higher the values of resources, the higher the values of extracted discriminant function, negative values meant that the higher the values of resources, the lower the values of extracted discriminant function.

**Figure 1:**
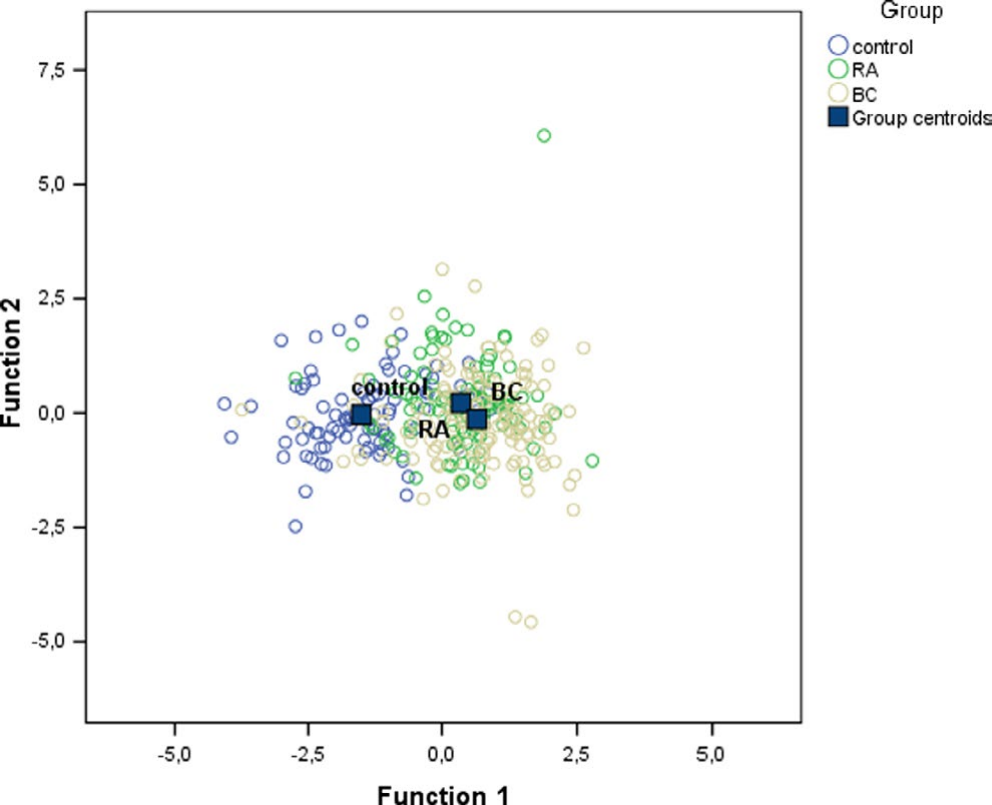
The values of the functions at group centroids in all study samples

The highest values of standardized coefficients in the first discriminant function were obtained for the level of hedonistic and vital resources and for the level of family resources. However, the value for hedonistic and vital resources was positive, while the value for family resources was negative, which indicates that group differences are explained by the difference between these two variables. Figure 1 presents the values of these functions at the group centroids. The first (and the only statistically significant) function differentiated between the control group and both clinical samples.

**Figure 2:**
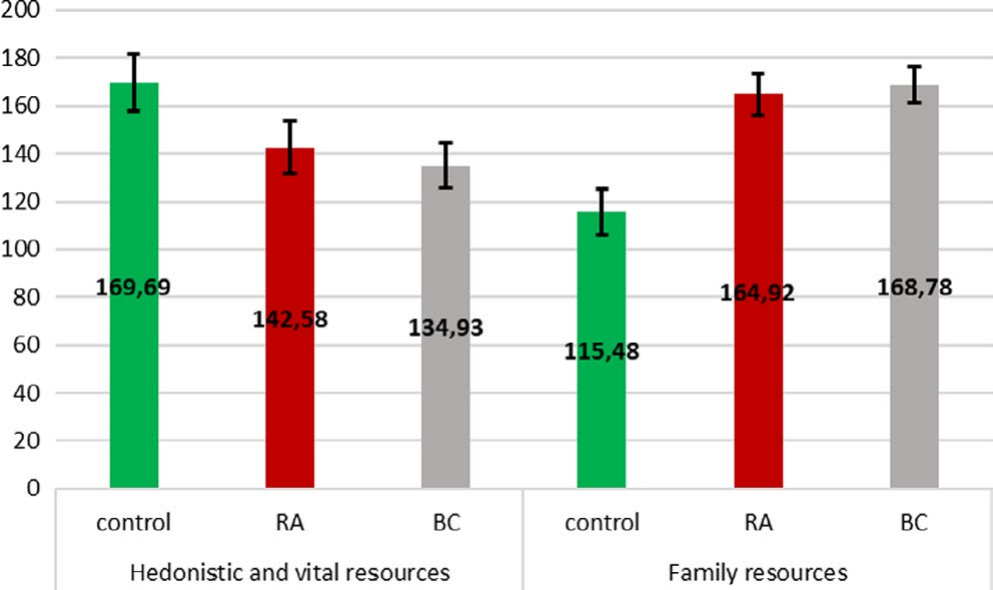
Profiles of hedonistic and vital resources and family resources in control group and clinical samples with 95% confidence intervals computed with the use of Bonferroni correction

The structure of the mean level of hedonistic and vital resources and family resources shows that the level of hedonistic and vital resources was higher in the control group, while the level of family resources was higher in the clinical groups, *η* ^2^ = .21, which supports Hypothesis 1 (see Figure 2). The RA and BC groups did not differ with regard to the levels of resources, which contradicts Hypothesis 2.

Hypothesis 3 and Hypothesis 4 were also verified using a discriminant analysis, but this time, body image dimensions and participants' ages were analyzed as independent variables. Table 4 presents the values of the standardized canonical discriminant function coefficients. According to Wilks's lambda test, the first function, *χ* ^2^(22) = 276.52, *p* < .001, was statistically significant, and the second function was statistically significant, *χ* ^2^(10) = 49.10, *p* < .001, which means only the first discriminant function discriminated between the analyzed groups.

**Table 4:** Standardized canonical discriminant function coefficients for body image scales

Body image	Function 1	Function 2
Appearance evaluation	−0.04	0.30
Appearance orientation	−0.33	−0.17
Fitness evaluation	0.02	0.30
Fitness orientation	0.16	0.60
Health evaluation	−0.59	−0.19
Health orientation	0.72	−0.33
Illness orientation	0.42	0.23
Overweight preoccupation	−0.08	0.32
Body areas satisfaction	0.06	0.37
Self‐classified weight scale	0.18	0.21
Age	0.93	−0.05

Note

Positive values of standardized canonical discriminant function mean that the higher the values of body image dimension, the higher the values of extracted discriminant function, negative values meant that the higher the values of body image dimension, the lower the values of extracted discriminant function.

**Figure 3:**
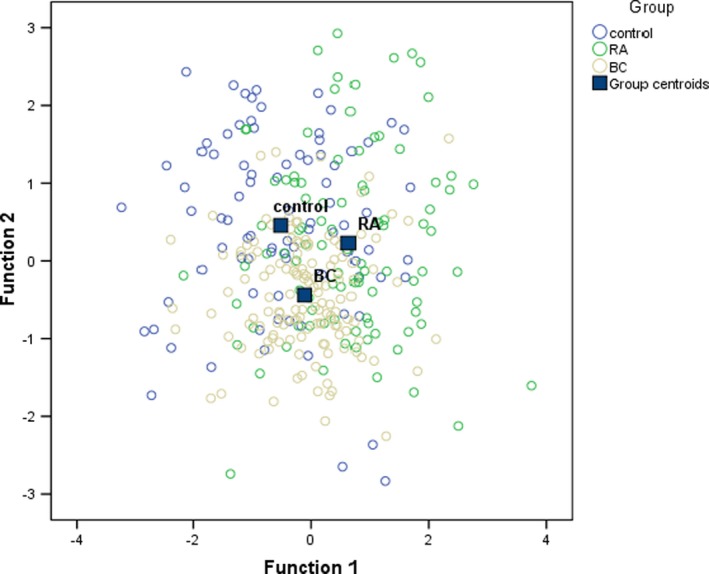
The values of the functions for body image at group centroids in all study samples

Besides participants' ages, the first function was that of the relationship between health orientation, orientation to the disease, and health assessment. The values of health orientation and orientation to the disease were positive, and the value of health assessment was negative, which indicates that the difference between health assessment and health orientation and that between health assessment and orientation to the disease accounted for the between‐group differences. The second function was the function of fitness orientation, satisfaction with the areas of the body, evaluation of appearance, fitness assessment, and preoccupation with weight. All values of these body dimensions were positive. Figure 3 presents the values of the functions at the group centroids. The first function differentiated between the control group and the RA group, and the second function differentiated between the BC group and both the control group and the RA group.

**Figure 4:**
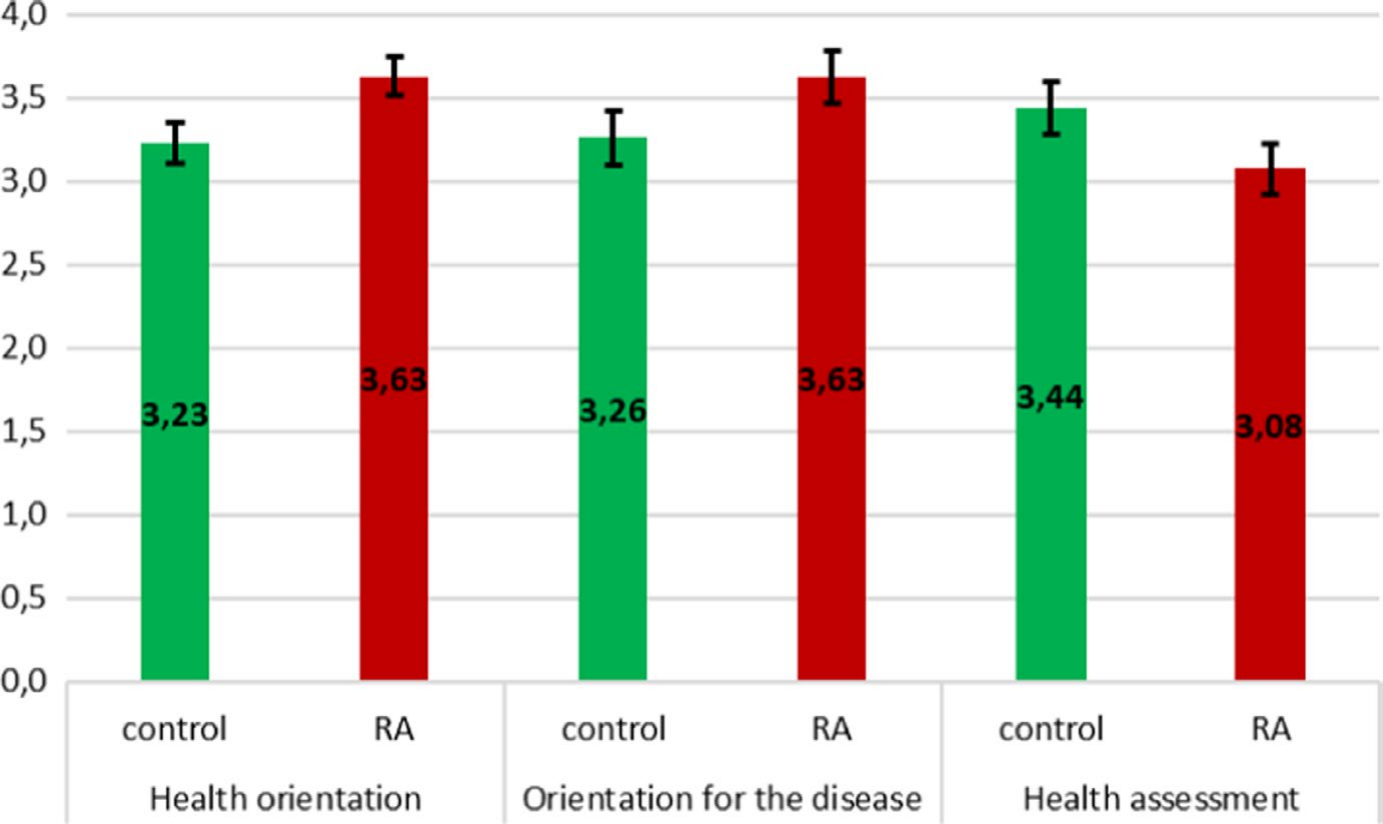
Profiles of health orientation, orientation for the disease, and health assessment in the control group and the RA groups with 95% confidence intervals computed with the use of Bonferroni correction

Figure 4 presents the mean values of health orientation, orientation to the disease, and health assessment in the control group and the RA group only. The acquired differences support Hypothesis 3, *η* ^2^ = .08.

**Figure 5:**
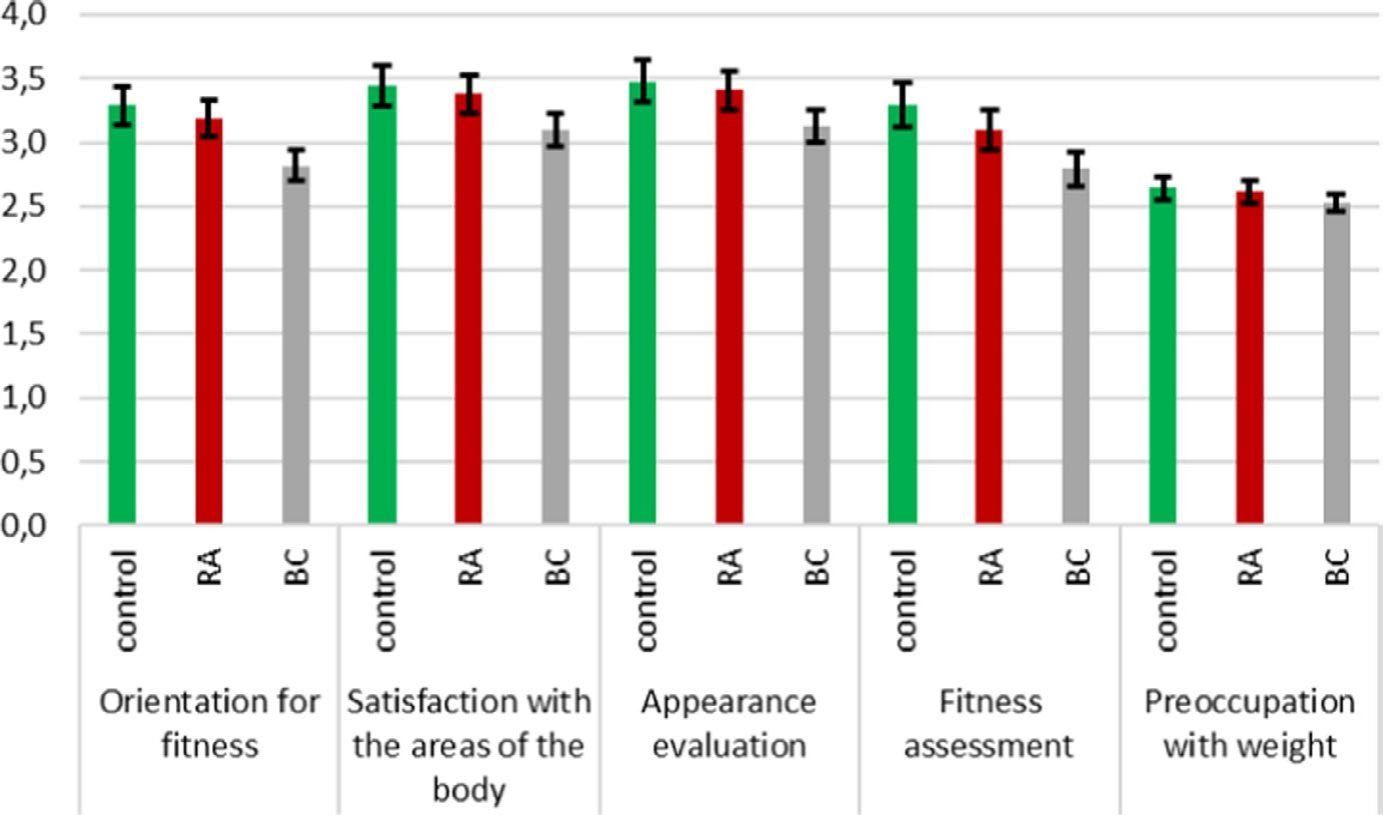
Profiles of fitness evaluation, satisfaction with the Aareas of the body, appearance orientation, fitness assessment, and preoccupation with weight in the control group, the RA sample and the BC sample

Figure 5 presents the mean values of fitness evaluation, fitness orientation, appearance evaluation, weight preoccupation, and body area satisfaction in the control group, the RA group, and the BC group. The means of all the aforementioned variables were lower in the BC group than in the RA group or the control group, *η* ^2^ = .06, and these differences are in line with Hypothesis 4.

## 4 DISCUSSION

The results of our study were in line with Hypothesis 1, as females from the clinical groups declared lower levels of vital and hedonistic resources but higher levels of family resources compared with those of the healthy control group. At the same time, we did not find any differences in the profiles of resources between the clinical samples, and thus, no confirmation of Hypothesis 2 was obtained. It seems that living with RA or BC may result in similar resource evaluations, and this finding is interesting in itself because no previous studies have been conducted on this topic thus far. However, in comparison with healthy individuals, RA patients and BC patients mainly experience a lack vigor and energy and problems related to the active pursuit of one's interests and goals or the search for pleasure or enjoyment. This latter result is especially prevalent in RA patients, for whom one of the central symptoms is chronic fatigue (Pollard, Choy, Gonzalez, Khoshaba, & Scott, 2006), which is strongly related to daily pain, sleep problems, and depression (Nicassio et al., 2012). Importantly, some authors have observed that females with RA report more fatigue when compared to men with RA and thus experience much higher rates of RA pain and functional limitations (Thyberg et al., 2009). Similarly, several studies have pointed to the prevalence of cancer‐related fatigue among females with BC, which may be observed during treatment (Ancoli‐Israel et al., 2006) but are especially observed after mastectomy (Bardwell et al., 2008).

In addition, experiencing the studied illnesses may increase the subjective importance of family relationships. This corresponds to several systematic reviews that show that one of the most important predictors for quality of life and illness adaptation among both RA patients (Sharpe, 2016) and BC patients (Mols, Vingerhoets, Coebergh, & Poll‐Franse, 2005) is satisfaction gained through close family relationships. More specifically, for females with RA or BC, the major sources of support stem from intimate relationships, as these diseases pose multidimensional problems to self‐esteem because they are especially associated with sexual problems (Essam et al., 2016; Pumo et al., 2012).

This latter observation was somewhat observed while confirming Hypothesis 3, which is related to body image profiles; in the clinical sample of RA, greater health and disease orientation were noticed, while in the control group, a more positive health evaluation was observed. In other words, it seems that females from the RA sample experienced their body image as a function of the illness's progression, which is in line with some recent studies (e.g., Boyington et al., 2015; Vinoski‐Thomas, Warren‐Findlow, & Webb, 2019). However, one of the most interesting but somewhat counterintuitive results deals with Hypothesis 4, which relates to the differences in body image profiles between RA females and BC females. Namely, compared with BC females, RA females declared a greater fitness evaluation and fitness orientation, higher weight preoccupation, increased body area satisfaction, and better appearance evaluation. Women with BC have a very distorted body image, as they perceive their bodies as incomplete, having had a symbol of femininity and sexuality (i.e., the breasts) taken away from them regardless of whether or not they have undergone mastectomy (Gumus et al., 2010). This state of constant insecurity, relating not only to their femininity but also to their very lives, is a great psychological burden (Helms et al., 2008; Shichen et al., 2018; Puigpinós‐Riera et al., 2018) and may be responsible for the very poor body satisfaction observed among the BC participants.

### 4.1 Strengths and limitations

This theory‐driven study is the first comparative research on resource evaluation to use the COR theory and body image between these two clinical samples with an additional healthy control group, which is its strength. However, we should bear in mind that the cross‐sectional design of the study makes it difficult to draw a causal conclusion from the obtained results. Specifically, due to the study's design, we were able to assess not resource loss nor resource gain over time among the participants, but only the current level of the subjectively assessed resources. In addition, the study samples differed substantially regarding many demographic variables. These differences are particularly visible regarding age between the healthy group and the clinical samples. Finally, we did not have data on the severity of the illnesses' progression among the clinical samples, and thus, medical variables were not controlled sufficiently in our research.

## 5 CONCLUSION

Despite these limitations, this study adds to the literature on the psychological aspects of living with RA or BC among women. It seems that females with RA or BC differ with respect to the subjectively assessed resources and body image evaluation when compared to healthy women. Therefore, psychological counselling specifically designed for RA and BC females may help these women restore these aspects of their identities that have been altered by their respective illnesses.

## DISCLOSURES

Disclosure of potential conflicts of interest: Author A declares that he has no conflict of interest. Author B declares that she has no conflict of interest. Author C declares that he has no conflict of interest.

## CONFLICT OF INTERESTS

None declared.

## ETHICAL APPROVAL

Research involving human participants and/or animals: All procedures performed in studies involving human participants were in accordance with the ethical standards of the institutional and/or national research committee and with the 1964 Helsinki Declaration and its later amendments.

## INFORMED CONSENT

Informed consent was obtained from all individual participants included in the study.

## Data Availability

Data are available upon the request.
